# The clinical value of [^18^F]-fluoro-ethyl-L-tyrosine PET ([^18^F]FET-PET) correlated with MRI in patients with functioning pituitary adenomas: an observational cohort study

**DOI:** 10.1007/s11102-025-01634-w

**Published:** 2026-03-07

**Authors:** Loren S. van der Hoeven, Siebren G. van Vugt, Tessa Timmers, Eva A.M. Heshof, Miou S. Koopman, Eleonora Aronica, Jantien Hoogmoed, Alberto M. Pereira, Elsmarieke van de Giessen, Dirk Jan Stenvers

**Affiliations:** 1 Amsterdam UMC, Department of Endocrinology and Metabolism, University of Amsterdam, Amsterdam Gastroenterology Endocrinology and Metabolism (AGEM), Amsterdam, Netherlands; 2 Pituitary Center Amsterdam, Amsterdam, The Netherlands; 3 European Reference Network on Rare Endocrine Conditions (Endo-ERN), Amsterdam, the Netherlands; 4 Department of Radiology & Nuclear Medicine, Amsterdam UMC, Vrije Universiteit, Amsterdam, the Netherlands; 5 Amsterdam UMC, Department of Radiology and Nuclear Medicine, University of Amsterdam, Amsterdam, the Netherlands; 6 Amsterdam UMC, Department of (Neuro)Pathology, University of Amsterdam, Amsterdam, The Netherlands; 7 Amsterdam UMC, Department of Neurosurgery, University of Amsterdam, Amsterdam, The Netherlands; 8 Amsterdam Neuroscience, Brain Imaging, Amsterdam, The Netherlands

**Keywords:** Functioning pituitary adenoma, Functional imaging, Adenoma localization, [^18^F]FET, PET, Cushing’s disease, Acromegaly

## Abstract

**Purpose:**

To assess the clinical value of [^18^F]fluoroethyl-L-tyrosine PET ([^18^F]FET-PET) correlated with MRI in patients with functioning pituitary adenoma (FPA) with negative or equivocal conventional MRIs during diagnosis, persistent disease and recurrence.

**Methods:**

Retrospective observational cohort study of 34 patients with FPAs who underwent a total of 37 [^18^F]FET-PETs (Cushing’s disease: *n* = 19, acromegaly: *n* = 14, prolactinoma: *n* = 3, and TSH producing adenoma: *n* = 1) between January 2022 and April 2025. The clinical performance was assessed in the surgically treated cohort, using confirmative histopathology and/or postoperative remission as reference standard.

**Results:**

[^18^F]FET-PET identified a single lesion in 28 scans (76%), two lesions in 1 scan (3%), and no lesion in 8 scans (22%). [^18^F]FET-PET and MRI were concordant positive in 14/37 scans, concordant negative in 1/37, discordant MRI+/[^18^F]FET-PET + different location in 2/37, discordant MRI-/[^18^F]FET-PET + in 8/37, discordant MRI+/[^18^F]FET-PET- in 7/37 and partly concordant in 5/37 scans. In 14 cases surgery resulted in confirmative histopathology and/or postoperative remission, 12 of those had a positive [^18^F]FET-PET. In 6 cases surgery did not result in confirmative histopathology and/or postoperative remission, of whom 5 had a positive [^18^F]FET-PET. Overall, the sensitivity was 86% and the positive predictive value 71%. In three patients with acromegaly, [^18^F]FET-PET was able to localize a lesion, despite biochemical control under continued somatostatin analogue treatment.

**Conclusion:**

[^18^F]FET-PET enhances lesion detection and improves personalized treatment in patients with FPA and negative or equivocal conventional MRIs throughout the disease course. Consensus on the timing of [^18^F]FET-PET with respect to medication and biochemical status is warranted.

**Supplementary Information:**

The online version contains supplementary material available at 10.1007/s11102-025-01634-w.

## Introduction

Functioning pituitary adenomas (FPAs) are rare endocrine conditions that cause diverse symptoms and clinical syndromes, affecting quality of life and life expectancy. The prevalence of FPAs ranges from 1 to 3 in 1.000.000 for thyrotropin secreting adenomas (TSH-oma) [1], 1–5 per 100.000 in Cushing’s disease (CD) [2], 2–6 in 100.000 in acromegaly [3], and 3–6 in 10.000 in prolactinomas [4]. Pituitary surgery is considered the primary treatment for most FPAs, including non-invasive microprolactinomas and well-encased macroprolactinomas [5–9]. Preoperative adenoma and remnant identification is essential for optimizing postoperative remission rates and reducing risks of surgical complications, such as hypopituitarism.

Conventional 1.5 Tesla (1.5T) or 3T magnetic resonance imaging (MRI) is standard of care to detect functional pituitary microadenomas, but has several limitations. MRI results can be negative or equivocal, which reaches up to 40% in CD [10, 11]. Technical refinements, such as dynamic perfusion series or 7 T MRI, may improve detection. However, these modalities are not widely available for clinical care, and result in higher detection rates of incidentalomas [12–14]. In CD, inferior petrosal sinus sampling (IPSS) is recommended when MRI fails to detect a lesion or the lesion is < 6 mm, to confirm diagnosis [6]. However, this is an invasive procedure and the positive predictive value (PPV) of lateralization is only around 70% [15–17]. Another challenge is to localize postoperative remnants due to their size, location, and the difficulty in distinguishing remnants from postoperative changes on MRI [18]. Furthermore, when multiple remnants are detected by MRI, assessing the activity of each remnant is crucial for optimal preoperative counseling.

Molecular (functional) imaging techniques have emerged as a potential important imaging adjunct in the diagnostic work-up of FPAs. [^11^C]methionine positron emission tomography ([^11^C]MET-PET), the most studied molecular imaging technique, uses radiolabeled methionine to localize areas of increased amino acid uptake. Studies using [^11^C]MET-PET and MRI reported improved preoperative adenoma localization in CD [19, 20], acromegaly [21], prolactinomas [22, 23], and TSH-oma [24]. Studies have also shown the applicability of [^11^C]MET-PET in persistent or recurrent acromegaly [21, 25, 26], CD [20, 27, 28], and prolactinomas [23], including a case of persistent TSH-oma [29]. However, the short half-life of [^11^C]MET (20 min) requires an on-site cyclotron, limiting its clinical availability. Additionally, sensitivity has not been superior to MRI in one study with a relatively high MRI sensitivity [30].

[^18^F]fluoroethyl-L-tyrosine (FET), an alternative amino acid tracer, has the main advantage of a longer half-life (110 min) and wider availability [31]. Clinical experience with [^18^F]FET-PET has been described in several small cohorts of preoperative and postoperative patients with CD [19, 32, 33], acromegaly [34], and prolactinoma [35], with sample sizes ranging from 2 to 22 cases (Table 1). There is a clear need for more pituitary reference centers to report their experiences.

**Table 1:** Current literature on the clinical performance of [^18^F]FET-PET in patients with functioning pituitary adenomas

Study	Population (size)	Disease stage	Biochemical activity	Medication	Results
Berkmann et al. (2021) [19]	Cushing’s disease ([^18^F]FET-PET: *n* = 7 [^18^F]FET-PET + [^11^C]MET-PET: *n* = 2)	Diagnosis (*n* = 7) History of previous TSS (*n* = 2)		Not reported	100% [^18^F]FET-PET positivity (concordant: *n* = 7, negative MRI: *n* = 2), 100% intraoperative and histological confirmation, 78% postoperative remission, 29% recurrence Sensitivity: 100% (95% CI 66.37–100) PPV: 100%
Slagboom et al. (2023) [32]	Cushing’s disease (*n* = 2)	Persistent (*n* = 1) Recurrence (*n* = 1)		Not reported	Persistent disease: [^18^F]FET-PET positive with negative MRI, adenoma localization confirmed by surgeon, no histopathological confirmation and no remission Recurrence: [^18^F]FET-PET positive, adenoma localization identified by surgeon, histopathological confirmation and postoperative remission.
Pruis et al. (2024) [33]	Cushing’s disease (*n* = 22), negative or inconclusive results on prior MRI	Diagnosis (*n* = 17) Recurrence (*n* = 5)		Pretreated with steroidgenesis inhibitors (*n* = 19) Cabergoline (*n* = 3) None of the patients used somatostatin analogs during [^18^F]FET-PET	100% [^18^F]FET-PET positivity (*n* = 6, partly concordant: *n* = 5, negative MRI: *n* = 11), 100% intraoperative confirmation by surgeon, 67% histological confirmation, pituicytoma *n* = 1, 80% postoperative remission during FU (median FU period 10 months (IQR, 4–16 months)) Sensitivity: 100% PPV: 73%
Bakker et al. (2024) [34]	Acromegaly (*n* = 10), persistent without a clear surgical target on MRI	Persistent disease after surgery (*n* = 5) or under medical therapy (*n* = 5)	Median last IGF-1 value before [^18^F]FET-PET/MRI^CR^: +3.7 SD (range 3.0–4.4)	At the time of [^18^F]FET-PET/MRI^CR^: - Pegvisomant (*n* = 8) - None (*n* = 2)	Persistent disease after surgery: 100% [^18^F]FET-PET/MRI^CR^ positive (concordant: *n* = 3, negative MRI: *n* = 2), 80% intraoperative and histological confirmation, 60% postoperative remission without complications Persistent disease under medical therapy: 100% [^18^F]FET-PET/MRI^CR^ positive (concordant: *n* = 2, partial concordant: *n* = 1, negative MRI: *n* = 2), 75% intraoperative and histological confirmation. 0% postoperative remission
van Trigt et al. (2024) [35]	Prolactinoma (*n* = 17), difficult-to-localize remnants	Persistent disease/recurrence Previous TSS (*n* = 9) Pre-treated with DA (*n* = 17)	Serum PRL levels closest to date of [^18^F]FET-PET/MRI^CR^ Median serum PRL: 3.6x ULN (range 1.0–20.3.0.3)	Pre-treated with DA: 100% DA were discontinued > 4 weeks before [^18^F]FET-PET/MRI^CR^ in all cases	82% [^18^F]FET-PET positivity (concordant: *n* = 9, negative MRI: *n* = 4, multifocal active lesion: *n* = 1), 62.5% histological confirmation, 62.5% remission

*[*^*11*^*C]-MET-PET/MRI*^*CR*^ [^11^C]methionine-PET co-registered with MRI, *DA* dopamine agonist, *[*^*18*^*F]FET-PET/MRI*^*CR*^ [^18^F]fluoroethyl-L-tyrosine-PET co-registered with MRI, *IGF-1* insulin-like growth factor 1, *MRI* magnetic resonance imaging, *PPV* positive predictive value, *PRL* prolactin, *SD* standard deviation, *TTS* transsphenoidal surgery, *x ULN* times upper limit of normal

The aim of this study is to describe our clinical results of [^18^F]FET-PET correlated with MRI, including diagnostic and therapeutic decision-making, in patients with any type of (suspected) FPA with a negative or equivocal MRI. The secondary aim is to determine the sensitivity and PPV in the surgically treated cohort using postoperative confirmative histopathology and/or biochemical remission as a reference standard.

## Patients and methods

### Patients

Retrospective cohort study of [^18^F]FET-PETs performed at the Amsterdam Pituitary Center in the period January 2022/April 2025 in patients with suspected or confirmed FPA. [^18^F]FET-PETs were excluded if no MRI for co-registration/correlation was available. All patients included provided informed consent for the reuse of medical data (2023.0438 and W23_025#23.049). Two included CD cases were described as exemplar cases in our review (#9 and 12) [32].

### Data collection

Baseline characteristics, previous diagnostic procedures, treatment modalities, histopathological results, and study parameters were extracted from the patients’ electronic health records.

#### Biochemical evaluation

Hormone levels (adrenocorticotropic hormone (ACTH), morning serum cortisol, insulin-like growth factor 1 (IGF-1), prolactin, free thyroxine (fT4) and thyroid-stimulating hormone (TSH)) and dynamic function tests (1 mg dexamethasone suppression tests (DST), 24-hour urinary free cortisol (UFC), late night salivary cortisol, 7 mg DST, CRH-test, IPSS, hair cortisol measurements, oral glucose tolerance tests (OGTTs), and cannulated stress-free sampling for prolactin) were collected at two time points: latest levels prior to [^18^F]FET-PET, and, if applicable, latest levels prior to start of pituitary-directed agents.

Diagnosis of FPA was made according to current international guidelines (Supplemental Table 1) [5–8, 36].

#### Indications for [^18^F]FET-PET

The multidisciplinary team (MDT) set all indications for [^18^F]FET-PET. The aim of the [^18^F]FET-PET was to localize active (secreting) adenoma tissue. The disease stages were classified as follows:

1. Diagnosis. De novo patients with biochemical evidence of hormonal hypersecretion of pituitary origin. MRI showed no lesion or was equivocal.
2. Persistent disease. Patients who did not achieve remission after surgery or treatment with medication.
3. Recurrence. Patients with recurrence following initial remission after surgery or treatment with medication.

### Imaging acquisition and analysis

#### MRI

MRI scans used for correlation were performed on 1.5T and 3.0T scanners at our center (Siemens and Philips), following our standard pituitary protocol, with a voxel size of 3 × 3 × 3 mm. This protocol included sagittal T1-weighted fat-saturated, coronal T2-weighted sequences, and sagittal and coronal T1-weighted sequences after intravenous contrast (Dotarem, dosage: 377 mg/mL, 0.2 mL/kg).

Dynamic series perfusion imaging and neuronavigation series (sagittal 3D T1 MPRAGE weighted, voxel size 0.9 × 0.9 × 0.9 mm; preoperatively) after intravenous contrast were added when clinically indicated. All MRIs were assessed by experienced neuroradiologists as part of routine clinical care. For study purposes, the first author (LH) reassessed all MRIs under supervision of one neuroradiologist (MK).

#### [^18^F]FET-PET image acquisition

Following a period of fasting (≥ 4 h), dynamic PET scans were acquired on either a highly sensitive large field of view PET/CT scanner (Quadra, Siemens), or a conventional PET/CT scanner (Ingenuity, Philips) from 0 to 40 min after intravenous injection of [^18^F]FET (Quadra: median 205 MBq [197–218], Ingenuity: median 208 MBq [200–211]). One [^18^F]FET-PET was acquired on a third PET/CT scanner (Vereos, Philips; 192 MBq). Participants’ heads were immobilized using a head band. Furthermore, head movement was checked during scanning using laser beams, and corrected if necessary. Standard corrections were performed, such as scatter, randoms, attenuation, decay and dead time.

#### [^18^F]FET-PET image analysis

[^18^F]FET-PET scans were initially visually assessed by an experienced nuclear radiologist (EvdG), using Hermia software (Hermes Medical Solutions, Stockholm, Sweden). These findings were compared to the conventional MRI, and, in 76% of the positive [^18^F]FET-PETs, co-registered with 3D T1 MPRAGE weighted series to improve anatomical correlation. The certainty of the visual read of [^18^F]FET-PET was scored using a Likelihood-scale from 1 to 5 (1: no lesion visible, 2: likely no adenoma visible, 3: possibly adenoma visible, 4: likely adenoma visible, 5: certainly adenoma visible).

In addition, semi-quantitative measures were added. For this purpose, the summation images from 20 to 40 min after intravenous injection of [^18^F]FET was used to manually draw a 5 mm^3^ volume of interest (VOI) in (1) the pituitary lesion(s), (2) the left and right cavernous sinus and (3) a 15 mm^3^ VOI in the left and right temporal lobe. Standardized Uptake Value (SUV) max, SUVpeak and SUVmean were extracted from these VOIs. Lesion to background ratio (LBR) was calculated by dividing the SUVmax in the lesion by the SUVmean of the left and right temporal lobe.

Interpretation of the scans was categorized as follows: concordant positive (MRI+/[^18^F]FET-PET + same location), concordant negative (MRI-/[^18^F]FET-PET-), discordant MRI+/[^18^F]FET-PET + different location, discordant MRI-/[^18^F]FET-PET+, discordant MRI+/[^18^F]FET-PET-, or partly concordant.

### MDT Meeting

The results of the [^18^F]FET-PET, the MRI and their correlation were reviewed by the MDT to provide an individualized treatment plan. Treatment strategies included surgical resection, possibly preceded or followed by medication and/or radiotherapy, bilateral adrenalectomy in case of CD, medication, radiotherapy, radiosurgery, or a wait-and-scan policy.

### Follow-up

Biochemical remission was evaluated within 3–6 months after surgery and routinely repeated during follow-up (Supplemental Table 1). Adenoma localization during surgery was documented. Tissue samples were assessed by an experienced neuropathologist (EA) and classified according to the WHO 2017 and 2022 classification.

### Statistical analysis

Data are described as proportions (%), mean ± SD for normally distributed data, and median [IQR: 1st - 3rd] or median (range) for non-normally distributed data. Sensitivity and PPV were calculated using standard 2 × 2 contingency tables. Mann-Whitney U and Kruskal-Wallis tests compared non-normally distributed data between groups. Spearman correlations assessed relationships between biochemical results and SUVmax, SUVpeak, LBR, and Likelihood-scales. P-values < 0.05 were considered statistically significant. Analysis were performed using IBM SPSS Statistics 28.0 (SPSS INC., Chicago, IL, USA).

## Results

### Overall cohort

**Fig. 1:**
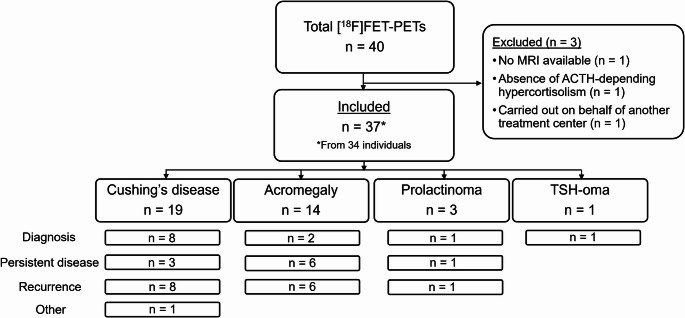
Flowchart of included [^18^F]FET-PETs

Forty pituitary [^18^F]FET-PETs from 37 individuals were conducted between January 2022/April 2025. Three [^18^F]FET-PETs were excluded: no MRI (*n* = 1), ACTH-independent hypercortisolism (*n* = 1), and request of [^18^F]FET-PET by another treatment center (*n* = 1). In total, 37 [^18^F]FET-PETs from 34 individuals with (suspected) CD (*n* = 19), acromegaly (*n* = 14), prolactinoma (*n* = 3), or TSH-oma (*n* = 1) were included, with a median follow-up duration of 1.0 year [0.6–1.8] (Fig. 1; Table 2). Median age at time of [^18^F]FET-PET was 45 years [35–58] and 68% of the scans were from females.

**Table 2:** Characteristics of the cases per [^18^F]FET-PET

	All (*n* = 37)	Cushing’s disease (*n* = 19)	Acromegaly (*n* = 14)	Prolactinoma (*n* = 3)	TSH-oma (*n* = 1)
**Age** **at** **[**^**18**^**F]FET-PET** **(years)**	45 [35–58]	45 [35–57]	42 [34–59]	38 (22–69)	79
**Sex** **(female)**	25 (68)	16 (84)	7 (50)	2 (67)	0 (0)
**Stage** **of** **disease** **at** **time** **of** **[**^**18**^**F]FET-PET**					
Diagnosis	12 (32)	8 (42)	2 (14)	1 (33)	1 (100)
Persistent disease	10 (27)	3 (16)	6 (43)	1 (33)	0 (0)
Recurrence	14 (38)	7 (37)	6 (43)	1 (33)	0 (0)
Other	1 (3)	1 (5)	0 (0)	0 (0)	0 (0)
**Results** **of** **MRI**					
No visible lesion	9 (24)	6 (32)	3 (21)	0 (0)	0 (0)
(Suspected) microadenoma (< 10 mm)	21 (57)	11 (58)	7 (50)	2 (67)	1 (100)
(Suspected) macroadenoma (≥ 10 mm)	3 (8)	1 (5)	2 (14)	0 (0)	0 (0)
Multiple lesions/remnants	4 (11)	1 (5)	2 (14)	1 (33)	0 (0)
**Surgical** **treatment** **prior** **to** **[**^**18**^**F]FET-PET**					
None	12 (32)	8 (42)	2 (14)	1 (33)	1 (100)
1 surgery	13 (35)	4 (21)	7 (50)	2 (67)	0 (0)
2 surgeries	9 (24)	5 (26)	4 (29)	0 (0)	0 (0)
3 surgeries	3 (8)	2 (11)	1 (7)	0 (0)	0 (0)
**Prior** **confirmed** **histopathological** **diagnosis**^a^					
Corticotroph adenoma	8 (32)	8 (73)	-	-	
Somatotroph adenoma	10 (40)	-	10 (83)	-	
Lactotroph adenoma	2 (8)	-	-	2 (100)	
Thyreotroph adenoma	0 (0)	-	-	-	0 (0)
Time between previous surgery and [^18^F]FET-PET^a^ (years)	4.2 [1.0–9.4]	4.1 [1.2–6.5]	4.7 [0.7–10.3]	9.1 (1.5–16.8)	-
**Treatment** **with** **pituitary-direct** s**agents**^b^ **during** **or** **within** **6** **months** **prior** **to** **[**^**18**^**F]FET-PET**					
None	26 (70)	19 (100)	5 (36)	1 (33)	1 (100)
Continued during [^18^F]FET-PET	8 (22)	0 (0)	6 (43)	2 (67)	0 (0)
Discontinued within 6 months prior to [^18^F]FET-PET	3 (8)	0 (0)	3 (21)	0 (0)	0 (0)
**Last** **known** **biochemical** **status** **at** **time** **of** **[**^**18**^**F]FET**-**PET**					
ACTH (pmol/L; ref 0–9)	-	11.0 [8.4–18.5]	-	-	-
2/3 abnormal hypercortisolism screening tests	-	14 (74)	-	-	-
Abnormal overnight 1 mg dexamethasone suppression test	-	17 (89)	-	-	-
Abnormal 24-hour urinary free cortisol	-	14 (74)	-	-	-
Abnormal late night salivary cortisol^c^	-	15 (88)	-	-	-
IGF-1 (SD; ref − 2.0–2.0)	-	-	3.0 [1.9–4.0]	0.3 (−0.8–0.8)	2.1
Prolactin (x ULN; ref ≤ 1)	-	-	0.5 [0.3–0.6]	3.0 (2.8–3.7)	0.5
fT4 (pmol/L; ref 12.0–22.0)	-	-	-	-	6.9
**End of** **study status**					
Under follow-up	30 (81)	16 (84)	12 (86)	2 (67)	0 (0)
Referred back to referral center	5 (14)	2 (11)	1 (7)	1 (33)	1 (100)
Lost to follow-up	2 (5)	1 (5)	1 (7)	0 (0)	0 (0)
Death	0 (0)	0 (0)	0 (0)	0 (0)	0 (0)
**Follow-up duration (years)**	1.0 [0.6–1.8]	1.3 [1.0–2.3]	0.7 [0.5–1.2]	0.5 (0.2–0.9)	0.2

Values are presented as number (percentage), median [IQR: 1st – 3rd], or median (range: minimum – maximum)

*ACTH* adrenocorticotropic hormone, *[*^*18*^*F]FET-PET* [^18^F]fluoroethyl-L-tyrosine-PET, *fT4* Free Thyroxine Hormone, *IGF-1* insulin-like growth factor 1, *MRI* magnetic resonance imaging, *SD* standard deviation, *TSH-oma* TSH-producing pituitary adenoma, *x ULN* times upper limit of normal

^a^ Proportions are calculated based on the number of patients that received at least one prior surgery

^b^ Pituitary-directed agents used in this cohort were somatostatin analogues (Octreotide and Lanreotide) and a dopamine receptor agonist (Cabergoline)

^c^ Late night salivary cortisol known in 17 patients

The [^18^F]FET-PETs were made during the diagnostic stage (*n* = 12), persistent disease (*n* = 10), recurrence (*n* = 14), and other (*n* = 1; suspected Nelson’s syndrome). MRI showed no visible lesion (*n* = 9), suspected microadenoma (*n* = 21), macroadenoma (*n* = 3), or multiple lesions (*n* = 4). Previous confirmative histopathology was present in the majority of [^18^F]FET-PETs of persistent disease or recurrence (CD: 73%, acromegaly: 83%, and prolactinoma: 100%). See supplemental Table 2 for detailed scan information.

### Indication for [^18^F]FET-PET

The indications for [^18^F]FET-PETs were to identify and localize an adenoma that was not seen on previous MRIs (*n* = 8), to confirm increased tracer uptake of suspected microadenoma during diagnosis (*n* = 7), of which 5 were smaller than 6 mm in CD, to confirm increased tracer uptake of a remnant (*n* = 5), to discriminate between active remnants and postoperative changes (*n* = 11), and to localize the (most) active lesion out of multiple lesions seen on MRI (*n* = 4). In one scan, the additional indication was to identify a possible second adenoma due to discrepant histopathological results of the previous surgery (gonadotroph adenoma in clinical acromegaly; #25). In one patient with suspected Nelson’s syndrome, [^18^F]FET-PET was made to assess stereotactic radiotherapy as treatment option (#19).

### Results of [^18^F]FET-PET co-registered/correlated with MRI

[^18^F]FET-PET identified a single lesion in 28 scans (76%), two lesions in 1 scan (3%), and no lesion in 8 scans (22%) (Supplemental Table 2). Median SUVmax and LBR in the positive [^18^F]FET-PETs was 2.8 [2.4–3.1] and 2.9 [2.4–3.2], respectively (Table 3). SUVmax of lesions was higher than the SUVmax of the left and right cavernous sinus in 20 scans (71%). Overall, the Likelihood-scale was 5 in 15 (41%) scans, 4 in 4 (11%) scans, 3 in 8 (22%) scans 2 in 2 (5%) scans, and 1 in 8 (22%) scans. Semi-quantitative measures and Likelihood-scale did not differ between the PET/CT scanners in positive [^18^F]FET-PETs (Supplemental Table 3).

**Table 3:** SUV and Likelihood-scales of positive [^18^F]FET-PETs

	All (*n* = 29)^a, b^	Cushing’s disease (*n* = 15)^b^	Acromegaly (*n* = 11)^a^	Prolactinoma (*n* = 3)	TSH-oma (*n* = 0)
SUVmax of lesion	2.8 [2.4–3.1]	2.8 [2.5–3.1]	2.6 [3.2–3.2]	3.0 (range 2.9–4.6)	N/A
SUVpeak of lesion	1.6 [1.3–1.9]	1.5 [1.3–1.9]	1.8 [1.4–2.1]	1.9 (range 1.6–1.9)	N/A
SUVmax cavernous sinus	2.6 ± 0.4	2.7 ± 0.4	2.5 ± 0.4	2.6 (range 2.0–3.5)	N/A
SUVpeak cavernous sinus	1.8 ± 0.3	1.8 ± 0.3	1.7 ± 0.3	1.7 (range 1.3–2.0)	N/A
SUVmean temporal lobes	1.0 ± 0.2	1.1 ± 0.2	1.0 ± 0.2	0.9 (range 0.8–1.0)	N/A
LBR	2.9 [2.4–3.2]	2.6 [2.2–3.1]	2.9 [2.4–3.2]	3.5 (range 3.0–5.3)	N/A
Likelihood-scale^c^	5.0 [3.0–5.0]	4.0 [3.0–5.0]	5.0 [5.0–5.0]	5.0 (range 3.0–5.0)	N/A

Data are presented as mean ± SD for normally distributed data and median [1st-3rd IQR] or median (range: minimum – maximum) for non-normally distributed data. Lesion to background ratio (LBR) was calculated by dividing the SUVmax by the SUVmean of the left and right temporal lobe

*LBR* Lesion to background (temporal lobe) ratio, *SUV* Standardized Uptake Value, *TSH-oma* TSH-producing pituitary adenoma

^a^ One scan from a patient with acromegaly showed two lesions

^b^ SUV values of one scan from a patient with CD could not be calculated due to technical reasons

^c^ The assessments of the [^18^F]FET-PETs were given a likelihood-scale: 1: no lesion visible, 2: likely no adenoma visible, 3: possibly adenoma visible, 4: likely adenoma visible, 5: certainly adenoma visible

[^18^F]FET-PET localized a lesion in 8 (89%) of the cases with a negative MRI. Positive [^18^F]FET-PETs were concordant with MRI in 14/29 scans, and discordant (different location) in 2/29 scans. [^18^F]FET-PET was partly concordant with MRI in five scans, where MRI showed possibly multiple lesions and [^18^F]FET-PET at least one but not all of these lesions in 4 scans, while [^18^F]FET-PET showed possibly multiple lesions and MRI at least one but not all of these lesions in 1 scan. A suggestive lesion on MRI followed by a negative [^18^F]FET-PET was seen in seven scans. In one case, the positive [^18^F]FET-PET location (Likelihood-scale 2) was concordant with a structure on MRI interpreted as an intercavernous sinus (#21). Co-registration with MRI to improve anatomical correlation was possible in 76% of the positive [^18^F]FET-PETs and 38% of the negative [^18^F]FET-PETs. In the surgical cohort, co-registration with MRI was performed in 75%.

### Multidisciplinary team meeting and proposed treatment strategies

All but five [^18^F]FET-PETs have been discussed during the MDT meeting. Surgery was recommended 17 times. Of these cases, 14 [^18^F]FET-PETs showed a visible lesion (concordant positive: *n* = 9, partly concordant: *n* = 1, discordant MRI-/[^18^F]FET-PET+: *n* = 4). Despite a negative [^18^F]FET-PET, the MDT was convinced of a pituitary origin of hormonal hypersecretion in three cases, and exploratory endoscopic surgery was offered (#2, 16, and 20).

Shared decision making between surgery and continuation of medication was recommended five times. The MDT advised against surgery 7 times. Continuation of medication was advised twice, once in a MRI-/[^18^F]FET-PET + patient due to cavernous sinus invasion (#32) and in another patient with MRI+/[^18^F]FET-PET- (#33). Shared decision making between continuation of medication with or without radiotherapy was offered to a patient after his second [^18^F]FET-PET (#25), as re-resection after the first positive [^18^F]FET-PET did not result in confirmative histopathology nor remission, and uptake seen on the second [^18^F]FET-PET showed potential cavernous sinus involvement.

A wait-and-scan strategy was advised in 3 cases: one of recurrent CD with MRI+/[^18^F]FET-PET- (#17), the case with suspected Nelson’s syndrome as no visible target lesion for radiotherapy was seen on MRI despite positive [^18^F]FET-PET (#19), and a case of recurrent acromegaly and increasing IGF-1 levels but normal OGTT (#31).

### Clinical performance of [^18^F]FET-PET

#### Full surgical cohort

**Fig. 2:**
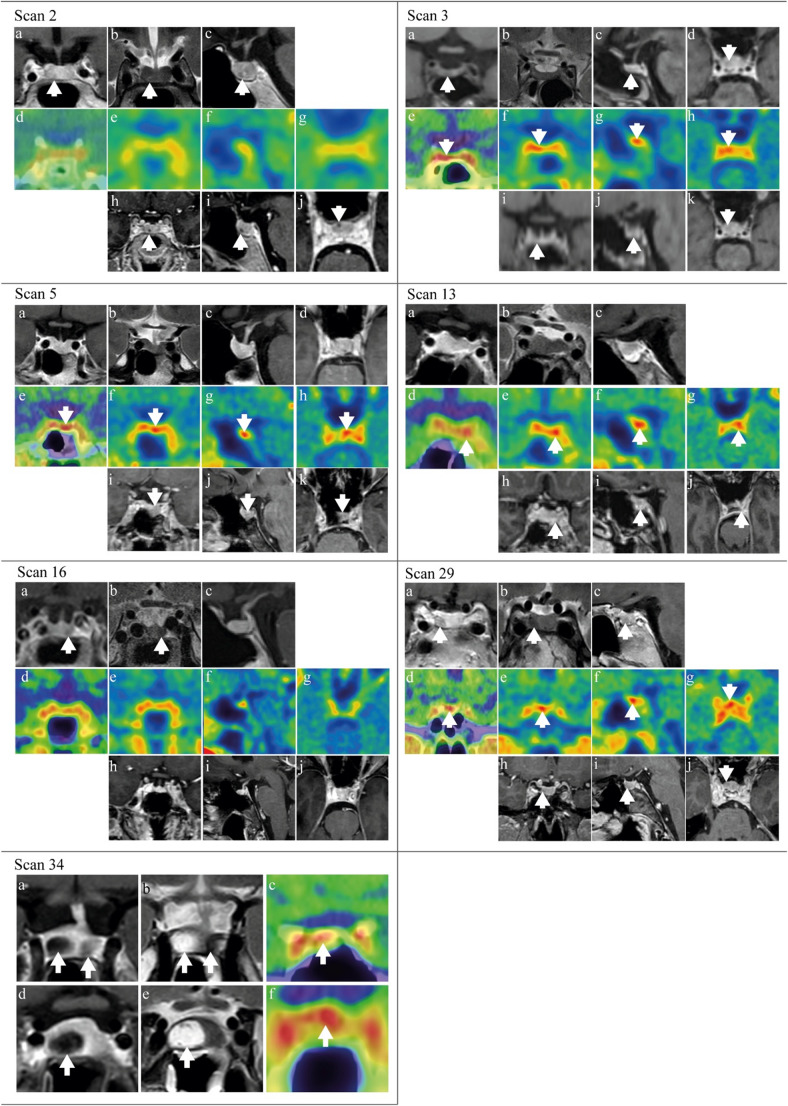
MRI co-registered/correlated with [^18^F]FET-PET images. **Scan 2:** Female patient in the diagnostic stage of Cushing’s disease (CD) with a lesion suspected for a microadenoma on conventional (**a**) coronal T1, (**b**) coronal T2 and (**c**) sagittal T1 post-contrast MRI. (**d**) [^18^F]FET-PET-CT image showed no focal uptake. Co-registration of (**e**) coronal, (**f**) sagittal, and (**g**) transversal [^18^F]FET-PET with (**h**) reconstructed 3D T1 coronal MPRAGE, (**i**) sagittal MPRAGE, and (**j**) reconstructed transversal MRPAGE. The patient underwent a TSS targeted at the lesion seen on MRI, which was located during surgery and suspected for adenoma tissue. Although no confirmative histopathology was present, patient reached postoperative remission and remained in remission until end-of-study. **Scan 3:** Female patient in the diagnostic stage of CD with a lesion suspected for a microadenoma on conventional 3T (**a**) coronal, and (**c**) sagittal T1 post-contrast MRI, and (**d**) 3D T1 reconstructed transversal MPRAGE, not visible on (**b**) coronal T2 post-contrast MRI. (**e**) [^18^F]FET-PET-CT image showed focal uptake right paramedian in the sella continuously until right cavernous sinus, concordant with the lesion localization on MRI. Co-registration of (**f**) coronal, (**g**) sagittal, and (**h**) transversal [^18^F]FET-PET with (**i**) 3D T1 reconstructed coronal MPRAGE, (**j**) sagittal MPRAGE, and (**k**) reconstructed transversal MRPAGE. Confirmative histopathology as well as postoperative remission were achieved after transsphenoidal surgery (TSS). **Scan 5:** Female patient in the diagnostic stage of CD without a circumscribed lesion visible on conventional 3T (**a**) coronal T1, (**b**) coronal T2, (**c**) sagittal T1, and (**d**) 3D T1 reconstructed transversal MPRAGE post-contrast MRI. (**e**) [^18^F]FET-PET-CT image showed focal uptake left paramedian in the sella. Co-registration of (**f**) coronal, (**g**) sagittal, and (**h**) transversal [^18^F]FET-PET with (**i**) 3D T1 reconstructed coronal MPRAGE, (**j**) sagittal MPRAGE, and (**k**) reconstructed transversal MRPAGE. In hindsight, the MRI showed a slightly asymmetrical sella as a secondary sign. Histopathology results showed preexistent pituitary tissue with a possible focus of a corticotroph microadenoma, however, this was not sufficient for a confirmative histopathological result, nor did the patient reach postoperative remission. A second TTS also did not result in remission and the patient was treated with bilateral adrenalectomy. **Scan 13:** Female patient with recurrent CD without a circumscribed lesion visible on conventional 3T (**a**) coronal T1, (**b**) coronal T2, and (**c**) sagittal T1 post-contrast MRI. (**d**) [^18^F]FET-PET-CT image showed focal uptake on the left side in the sella. Based on the localization of the primary adenoma this was suspected for an active remnant. Co-registration of (**e**) coronal, (**f**) sagittal, and (**g**) transversal [^18^F]FET-PET with (h) 3D T1 reconstructed coronal MPRAGE, (**i**) sagittal MPRAGE, and (**j**) reconstructed transversal MRPAGE. Despite the confirmative histopathology, the patient did not reach remission. **Scan 16:** Female patient with recurrent CD with a suspected remnant on the left side of the sella on conventional 1.5T (**a**) coronal T1 and (**b**) coronal T2 post-contrast MRI, not visible on (**c**) sagittal T1 post-contrast MRI. (**d**) [^18^F]FET-PET-CT image showed no focal uptake. Co-registration of (**e**) coronal, (**f**) sagittal, and (**g**) transversal [^18^F]FET-PET with (**h**) 3D T1 reconstructed coronal MPRAGE, (**i**) sagittal MPRAGE, and (**j**) reconstructed transversal MRPAGE. The patient underwent a TSS. During surgery, adenoma suspected tissue was seen on the right side. Although no confirmative histopathology was present, patient reached postoperative remission and remained in remission until end-of-study.** Scan 29:** Female patient in the stage of recurrent acromegaly with a lesion suspected of a remnant on conventional 3T (**a**) coronal, (**b**) coronal T2, and (**c**) sagittal T1 post-contrast MRI. (**d**) [^18^F]FET-PET-CT image showed focal uptake on the right side of the sella, concordant with the MRI location. Co-registration of (**e**) coronal, (**f**) sagittal, and (**g**) transversal [^18^F]FET-PET with (**h**) 3D T1 reconstructed coronal MPRAGE, (**i**) sagittal MPRAGE, and (**j**) reconstructed transversal MRPAGE. Patient underwent TSS that resulted in confirmative histopathology and postoperative remission. **Scan 34:** Female patient in the diagnostic stage of prolactinoma with two visible lesions, one of which partly cystic, on conventional 3T (**a**) coronal T1, and (**b**) coronal T2 post-contrast MRI. (**c**) [^18^F]FET-PET-CT image showed focal uptake on the right side of the sella, concordant with solid component of the cystic adenoma. (**d**) coronal T1, (**e**) coronal T2 post-contrast MRI and (**f**) [^18^F]FET-PET-CT focused on the right lesion. Patient was treated with TSS which resulted in both confirmative histopathology and postoperative remission

In total, 20 surgeries were performed (CD: 16/19, acromegaly 3/14, prolactinoma 1/3, TSH-oma: 0/1) (Table 4, Supplemental Table 4). Surgery was performed after 17 positive and 3 negative [^18^F]FET-PETs. Exemplar scans are shown in Fig. 2. One patient received surgery twice, once after every [^18^F]FET-PET (#11 and 14). The other two patients that underwent two [^18^F]FET-PETs were only surgically treated after their first [^18^F]FET-PET (#13 and 23). Suspected adenoma tissue was identified intraoperatively by neurosurgeons in all cases. In surgeries performed after positive [^18^F]FET-PETs the adenoma was located at the location of [^18^F]FET uptake in 88% and more lateral in the other two cases.

**Table 4:** [^18^F]FET-PET results and surgical outcomes

		**Diagnostic stage**				**Persistent disease/recurrence**		
**Total cohort**	**All** (*n* = 37)	**Cushing’s** **disease** (*n* = 8)	**Acromegaly** (*n* = 2)	**Prolactinoma** (*n* = 1)	**TSH-oma** (*n* = 1)	**Cushing’s** **disease** (*n* = 11)	**Acromegaly** (*n* = 12)	**Prolactinoma** (*n* = 2)
**[**^**18**^**F]FET-PET** **results**								
Concordant positive MRI+/[^18^F]FET-PET + same location	14 (38)	2 (25)	0 (0)	0 (0)	0 (0)	5 (45)	5 (42)	2 (100)
Concordant negative (MRI-/[^18^F]FET-PET-)	1 (3)	1 (13)	0 (0)	0 (0)	0 (0)	0 (0)	0 (0)	0 (0)
Discordant, MRI+/[^18^F]FET-PET + different location	2 (5)	2 (25)	0 (0)	0 (0)	0 (0)	0 (0)	0 (0)	0 (0)
Discordant, MRI+/[^18^F]FET-PET-	7 (19)	1 (13)	1 (50)	0 (0)	1 (100)	2 (18)	2 (17)	0 (0)
Discordant, MRI-/[^18^F]FET-PET+	8 (22)	1 (13)	1 (50)	0 (0)	0 (0)	4 (36)	2 (17)	0 (0)
Partly concordant, MRI shows multiple lesions, [¹⁸F]FET-PET not all	4 (11)	1 (13)	0 (0)	1 (100)	0 (0)	0 (0)	2 (17)	0 (0)
Partly concordant, [¹⁸F]FET-PET shows multiple lesions, MRI not all	1 (3)	0 (0)	0 (0)	0 (0)	0 (0)	0 (0)	1 (8)	0 (0)
		**Diagnostic stage**				**Persistent disease/recurrence**		
**Surgical** **cohort**	**All** (*n* = 20)	**Cushing’s ** **disease ** (*n* = 8)	**Acromegaly ** (*n* = 0)	**Prolactinoma ** (*n* = 1)	**TSH-oma ** (*n* = 0)	**Cushing’s ** **disease ** (*n* = 8)	**Acromegaly ** (*n* = 3)	**Prolactinoma ** (*n* = 0)
**PA** **results** **from first surgery after** **[¹⁸F]FET-PET**								
Positive	8 (40)	2 (25)		1 (100)		3 (38)	2 (67)	
Negative/inconclusive	12 (60)	6 (75)		0 (0)		5 (63)	1 (33)	
**PA** **results from later surgeries after [¹⁸F]FET-PET**								
Positive		0 (0)				1 (50)		
Negative/inconclusive		2 (100)				1 (50)		
**Biochemical remission 3–6 months after first surgery**								
3–6 months postoperative after first surgery after [^18^F]FET-PET	13 (65)	6 (75)		1 (100)		4 (50)	2 (67)	
**Impact of** **[**^**18**^**F]-FET-PET**								
Positive [^18^F]FET-PET followed by confirmative PA results^a^	8 (44)	2 (33)		1 (100)		3 (43)	2 (67)	
Positive [^18^F]FET-PET followed by confirmative PA results and/or postoperative remission without medication^a^	12 (67)	5 (83)		1 (100)		4 (57)	2 (67)	
Negative [^18^F]FET-PET with confirmative PA results^b^	0 (0)	0 (0)				0 (0)		
Negative [^18^F]FET-PET with confirmative PA results and/or postoperative remission without medication^b^	2 (67)	1 (50)				1 (100)		

Values are presented as number (percentage)

*[*^*18*^*F]FET-PET* [^18^F]fluoroethyl-L-tyrosine-PET, *MRI* magnetic resonance imaging, *TSH-oma* TSH-producing pituitary adenoma, *PA* histopathology

^a^ Proportions are calculated based on the number of patients with a positive [^18^F]FET-PET that received a surgery after the [^18^F]FET-PET

^b^ Proportions are calculated based on the number of patients with a negative [^18^F]FET-PET that received a surgery after the [^18^F]FET-PET

Of the 14 surgeries that resulted in confirmative histopathology and/or remission, 12 [^18^F]FET-PETs were positive (median Likelihood-scale 4.5 [3.3–5.0]). Of the six surgeries that did not result in confirmative histopathology and/or remission, 5 [^18^F]FET-PETs were positive (median Likelihood-scale 3.0 [2.5–5.0]). Thus, among patients with FPAs with negative or equivocal MRI, the overall sensitivity for localization of functional adenoma was 86% and the PPV 71%, with an overall specificity of 17% and negative predictive value (NPV) of 33%. In CD, the sensitivity was 82% and PPV 69%. The LBR and Likelihood-scale of positive [^18^F]FET-PETs did not differ between surgeries with and without confirmative histopathology and/or remission (*p* = 0.171, *p* = 0.308, respectively).

#### Diagnostic disease stage

The sensitivity in the diagnostic stage was 86% with a PPV of 86%, a specificity of 50% and NPV of 50%. Within the subgroup of CD, the sensitivity was 83%, PPV was 83% with a specificity of 50% and NPV of 50%.

#### Persistent disease/recurrence

In persistent disease and recurrence, the sensitivity was 86% and the PPV 60% with a specificity of 0% and NPV of 0%. Within the subgroup of CD, the sensitivity was 86% and the PPV 60% with a specificity of 0% and NPV of 0%.

### Medication prior to and at the time of [^18^F]FET-PET

Pituitary-directed agents were not used during, or used more than 6 months prior to, [^18^F]FET-PET in 26 (70%) scans, continued during 8 (22%) scans, and discontinued within 6 months prior to [^18^F]FET-PET in 3 (8%) scans. Use of pituitary-directed agents did not alter the proportion of positive [^18^F]FET-PETs between these groups (*p* = 0.237), and LBR and Likelihood-scale in positive [^18^F]FET-PETs did not differ (*p* = 0.098, *p* = 0.566, respectively) (Supplemental Table 5).

In CD patients, the only adrenal steroidogenesis inhibitor used was metyrapone, which did not lead to normalization of the 24 h UFC in any of the 3 cases. In acromegaly patients, somatostatin analogues (Octreotide and Lanreotide) with or without a GH receptor antagonist (Pegvisomant), or a dopamine receptor agonist (Cabergoline) were used at time of the [^18^F]FET-PET in six patients. Despite this use, and biochemical control in three patients (#21, 22, 28), all had positive [^18^F]FET-PETs (Likelihood-scales 2, 5 and 5). The uptake was assessed with a Likelihood-scale of 5 in all but one due to the concordance with the MRI of a structure interpreted as an intercavernous sinus (#21). Two prolactinoma patients used Cabergoline at time of the [^18^F]FET-PET, one without achieving biochemical control.

### Biochemical activity

Overall, the proportion of positive [^18^F]FET-PETs did not differ between patients with and without confirmed biochemical hypersecretion at the latest assessment before [^18^F]FET-PET (*p* = 0.446), neither did the median LBR and Likelihood-scale in positive [^18^F]FET-PETs (*p* = 0.248, *p* = 0.927) (Supplemental Table 5). Time between latest biochemical assessment and [^18^F]FET-PET did not differ between positive and negative [^18^F]FET-PETs (median 3.0 months [1.0–5.2] vs. 3.5 [1.0–4.1] (*p* = 1.000)). No correlations were found between latest 24 h UFC values in CD, IGF-1 levels in acromegaly and PRL levels in prolactinoma and LBR and Likelihood-scale in positive [^18^F]FET-PETs (Supplemental Table 6).

Biochemical hypersecretion at latest assessment before [^18^F]FET-PET in CD patients was confirmed in 17 (89%) scans (#19 excluded (median 4.3 months [2.5–8.3] before [^18^F]FET-PET). In one case of cyclic CD, supported by excessive hair cortisol levels, screening tests before [^18^F]FET-PET normalized (#15, 1.5 months before [^18^F]FET-PET). However, [^18^F]FET-PET revealed focal asymmetric uptake. (Likelihood-scale 4). Last documented IGF-level was increased in 10/14 (71%) scans of acromegaly patients (median 1.5 months [0.0–4.0] before [^18^F]FET-PET). Last documented prolactin level was increased in all three prolactinoma patients (median 3.0 months (range 1.0–3.0) before [^18^F]FET-PET). Last documented fT4 level in TSH-oma patient was decreased under thiamazole (1.0 month before [^18^F]FET-PET).

### Follow-up

Of the surgically treated patients, 13 patients (65%) reached remission after transsphenoidal surgery and one under medication (Supplemental Table 4). Bilateral adrenalectomy was performed in two patients with a primary diagnosis of CD and in one patient with recurrent CD. Recurrence was seen in one primary and one recurrent CD. A case with suspected recurrent CD and negative [^18^F]FET-PET (#17) obtained spontaneous biochemical remission during follow-up. It was ultimately concluded that the abnormal screening tests were caused by severe psychosocial stress.

## Discussion

In this study, conducted at a national center of expertise and Reference Center of the European Reference Network on Rare Endocrine Conditions, we evaluated the clinical value of [^18^F]FET-PET in the largest cohort to date of patients with FPAs and negative or equivocal MRIs, using real-world data from clinical practice. We included 37 [^18^F]FET-PETs from 34 individuals with CD, acromegaly, prolactinoma or TSH-oma, across all disease stages (diagnostic, persistent disease and recurrence). Overall, 78% of [^18^F]FET-PETs localized a lesion, concordant with MRI localization in 14 scans (67%), discordant (different location) in 2 scans (10%) and partly concordant in 5 scans (24%). Notably, in patients with negative MRIs, [^18^F]FET-PET identified lesions in 89%. In the surgically treated cohort (twenty post-[^18^F]FET-PET surgeries) sensitivity was 86% and PPV 71%, using confirmative histopathology and/or postoperative remission as a reference standard. In CD, the sensitivity was 82% and PPV 69%.

Our findings demonstrate that [^18^F]FET-PET contributes to adenoma localization and personalized treatment throughout the disease course. Furthermore, [^18^F]FET-PET complements conventional diagnostic modalities in several indications. Specifically in diagnosing CD, where current guidelines require IPSS for lesions smaller than 6 mm on MRI, [^18^F]FET-PET additionally provides superior anatomical localization compared to IPSS [19, 33], which has inadequate reliability for lateralization [6]. The sensitivity and PPV in the diagnostic stage were 86% in the surgically treated cohort, and 83% in the subgroup of surgically treated CD.

In persistent disease and recurrence, the sensitivity was 86% and the PPV 60% based on the surgical cohort. Within the subgroup of CD, the sensitivity was 86% and the PPV 60%. In persistent disease, [^18^F]FET-PET showed asymmetric uptake in all cases and therefore showed potential ability to localize remnants with increased uptake and distinguish these from postoperative lesions. The estimation of a complete resection likelihood in preoperative planning is key in this group as prior surgery failed to achieve remission. Mainly in persistent disease, the MDT advised against surgery in some cases. Reasons were the absence of a radiological substrate or low likelihood of curative resection, such as cavernous sinus involvement identified by [^18^F]FET-PET, specifically in combination with tumor consistency based on previous surgery/surgeries and postoperative scarring and adhesions. Importantly, most patients with persistent or recurrence acromegaly or prolactinoma opted for medication despite surgical options. Thus, while [^18^F]FET-PET is valuable for preoperative planning and informed decision-making, patient preferences should be considered to maintain a balanced costs-benefit ratio. In recurrence, [^18^F]FET-PET showed asymmetrical uptake in patients with negative MRI findings, as well as in most patients with lesions on MRI. When used to confirm increased tracer uptake of MRI positive suspected remnants, [^18^F]FET-PET demonstrated focal uptake in all cases. In contrast, when MRI was equivocal and [^18^F]FET-PET was used to distinguish possible adenoma tissue from postoperative changes, [^18^F]FET-PET was positive in only 50%.

[^18^F]FET-PET also improved presurgical planning in patients with multiple lesions or remnants on MRI, as exemplified by scan 8, 22, 27 and 34. In untreated patients, [^18^F]FET-PET showed increased uptake only in the biochemically active lesions, distinguishing these lesions from biochemically inactive lesions (#8 and 34), allowing targeted surgery and avoided unnecessary exploration. Confirmative histopathology and postoperative remission were achieved in both cases without additional pituitary hormone deficiencies. In a patient with persistent acromegaly (#27), the recommendation of the MDT was impacted by [^18^F]FET-PET showing uptake in the sellar remnant, whilst ruling out extrasellar uptake, and surgery could be offered.

For surgically treated CD patients, sensitivity was 82% and PPV 69% based on 16 post-[^18^F]FET-PET surgeries. During diagnosis, sensitivity and PPV were slightly higher (both 83%), and during recurrence, both were 80%. Previous studies reported a sensitivity of 100% and PPV of 73–100% for [^18^F]FET-PET in CD, though Berkmann et al. (2021) included only patients with histopathologically proven corticotroph adenoma [19, 33]. In our study, [^18^F]FET-PET did not reveal a lesion in 21% of CD patients. Remarkably, 2 of 3 patients with negative [^18^F]FET-PET but suspected MRI lesions who underwent surgery achieved postoperative remission, albeit without confirmative histopathology. These patients had biochemical evidence of hypersecretion at last assessment and did not show signs of cyclic CD. This showcases that, in selected cases, explorative endoscopic transsphenoidal surgery targeting MRI identified lesions remains justified despite negative [^18^F]FET-PET. The lower overall PPV in our study compared to literature [19, 33] may reflect inclusion of patients with persistent disease, who have reduced likelihood of confirmative histopathology or remission after redo surgery.

In acromegaly, only three patients underwent surgery post-[^18^F]FET-PET, precluding calculation of sensitivity and specificity. [^18^F]FET-PET showed focal uptake in 71% of scans, but not in three [^18^F]FET-PETs during diagnostic and recurrence disease stage. To our knowledge, [^18^F]FET-PET results have not been reported during these disease stages. Two patients with negative [^18^F]FET-PETs had mildly elevated IGF-1 at last assessment without medication; the third had IGF-1 just below the ULN after skipping one Lanreotide dose, which could explain the negative scan. Bakker et al. (2024) described focal uptake in 100% of [^18^F]FET-PETs in persistent acromegaly, which is consistent with our findings [34].

In prolactinoma patients, all with hyperprolactinaemia at last assessment, [^18^F]FET-PET showed focal uptake in all cases. The surgically treated patient achieved postoperative remission with confirmative histopathology. van Trigt et al. (2024) described the utility of [^18^F]FET-PET/MRI^CR^ in complex prolactinomas and showed focal uptake in 82% of [^18^F]FET-PET/MRI^CR^, which influenced clinical decision-making in 88% of cases. Confirmative histopathology and postoperative remission was achieved in 63% [35].

There is currently no guideline or consensus statement to standardize timing of [^18^F]FET-PET relative to medication use and biochemical activity. As such, and due to the retrospective design of our study, use of medication and biochemical activity during [^18^F]FET-PET was not standardized in our cohort. However, this revealed some important findings. As hypothesized, use of metyrapone did not result in negative [^18^F]FET-PETs. Strikingly, somatostatin analogues, despite biochemical control in some cases, and dopamine receptor agonist cabergoline during [^18^F]FET-PETs did not result in negative scans either, despite their targeted inhibitory effects on GH and prolactin secretion, respectively. LBR and Likelihood-scale in positive [^18^F]FET-PETs did not differ significantly between patients on pituitary-directed agents and those without. We hypothesize that pituitary-directed agents may suppress the uptake of [^18^F]FET on the individual level. Potentially, subtraction PET images with and without the use of pituitary-directed agents may reveal an area of change, which may be helpful in cases with negative [^18^F]FET-PET results [24]. Practical planning aspects of [^18^F]FET-PETs led to prolonged time between [^18^F]FET-PET and latest biochemical assessment, although this was not different between positive and negative [^18^F]FET-PETs. Although there was no clinical suspicion of cyclic disease, causality between biochemical inactivity at scan time and negative scan result cannot be excluded. Nevertheless, [^18^F]FET-PET showed focal uptake in 75% of acromegaly patients with biochemical control. There were no differences in LBR and Likelihood-scale in positive [^18^F]FET-PETs between patients with and without biochemical hypersecretion at latest assessment. No correlations were found between LBR and Likelihood-scale and 24 h UFC in CD, IGF-1 in acromegaly and PRL in prolactinoma. Effect of medical therapy and timing between latest biochemical assessment needs to be studied further, as this is the only study to our knowledge where somatostatin analogues or dopamine receptor agonists were continued.

Our study has several limitations, mainly caused by the retrospective study design. First, the lack of standardized protocols on [^18^F]FET-PET timing, medication use and biochemical assessment limits our interpretation of negative [^18^F]FET-PET reliability. Additionally, real-world clinical practice also prevented co-registration in some [^18^F]FET-PETs. Instead, some [^18^F]FET-PETs were correlated with conventional MRI, potentially decreasing adenoma localization precision for pre-surgical planning. MRIs used for co-registration or correlation were not simultaneously made with the [^18^F]-FET-PET, which could lead to a prolonged period between [^18^F]-FET-PET and MRI. Compared to [^11^C]-MET, [^18^F]-FET is an amino acid analogue instead of an essential amino acid, which may result in washout of [^18^F]-FET in the cavernous sinus, as observed by Bakker et al. (2024) [34]. The impact on interpretation of possible lesions near or invading the cavernous sinus remains unclear and needs to be addressed in future studies. Also, as confirmative histopathology and/or postoperative remission were used as reference standards, only surgically treated patients were included in performance analysis. On the other hand, we included postoperative remission to the reference standard to reduce false positives, as total hypophysectomy is not ethical and tissue analysis of microadenomas may not always yield representative samples (sampling bias). False positives based on heterogenous physiological uptake could not be completely ruled out, but it is less likely that postoperative changes resulted in false positives as the median time between a previous surgery and the [^18^F]FET-PET was 4.2 years. Besides, this study was performed in a tertiary referral center with experienced MDT members, and, specifically, highly experienced neurosurgeons. Patients were selected based on existing hurdles in daily clinical practice with conventional diagnostic modalities, and we included patients with all types of FPAs, resulting in the largest cohort of [^18^F]FET-PETs assessments that is currently, to our knowledge, described in literature.

In conclusion, [^18^F]FET-PET improves adenoma detection and localization throughout the disease course in patients with FPAs and negative, equivocal or difficult-to-interpret MRI findings. Consensus on optimal timing of [^18^F]FET-PET relative to medication and biochemical status is warranted, particularly to aid interpretation and significance of negative [^18^F]FET-PET results. We recommend referral to national or international centers of expertise (i.e. European Reference Centers for Rare Endocrine Conditions), as both [^18^F]FET-PET assessment and surgical treatment requires the expertise of an experienced multidisciplinary team.

## Supplementary information

Below is the link to the electronic supplementary material.

ESM 1 (DOCX 72.4 KB)

## Data Availability

No datasets were generated or analysed during the current study.

## References

[1] Ónnestam L, Berinder K, Burman P, Dahlqvist P, Engström BE, Wahlberg J et al (2013) National incidence and prevalence of TSH-secreting pituitary adenomas in Sweden. J Clin Endocrinol Metab 98(2):626–35 23295463 10.1210/jc.2012-3362

[2] Giuffrida G, Crisafulli S, Ferraù F, Fontana A, Alessi Y, Calapai F et al (2022) Global cushing’s disease epidemiology: a systematic review and meta-analysis of observational studies. J Endocrinol Invest 45(6):1235–1246 35133616 10.1007/s40618-022-01754-1

[3] Kerbel J, Cano-Zaragoza A, Espinosa-Dorado R, de la García Torre KE, Mercado M (2023) Real world data on the epidemiology, diagnosis, and treatment of Acromegaly: a registries-based approach. Arch Med Res 54(6):102856 37481822 10.1016/j.arcmed.2023.102856

[4] Chanson P, Maiter D (2019) The epidemiology, diagnosis and treatment of Prolactinomas: the old and the new. Best Pract Res Clin Endocrinol Metab 33(2):101290 31326373 10.1016/j.beem.2019.101290

[5] Beck-Peccoz P, Lania A, Beckers A, Chatterjee K, Wemeau JL (2013) 2013 European thyroid association guidelines for the diagnosis and treatment of thyrotropin-secreting pituitary tumors. Eur Thyroid J 2(2):76–82 24783044 10.1159/000351007 PMC3821512

[6] Fleseriu M, Auchus R, Bancos I, Ben-Shlomo A, Bertherat J, Biermasz NR et al (2021) Consensus on diagnosis and management of Cushing’s disease: a guideline update. Lancet Diabetes Endocrinol 9(12):847–75 34687601 10.1016/S2213-8587(21)00235-7 PMC8743006

[7] Katznelson L, Laws ER Jr., Melmed S, Molitch ME, Murad MH, Utz A et al (2014) Acromegaly: an endocrine society clinical practice guideline. J Clin Endocrinol Metab 99(11):3933–51 25356808 10.1210/jc.2014-2700

[8] Petersenn S, Fleseriu M, Casanueva FF, Giustina A, Biermasz N, Biller BMK et al (2023) Diagnosis and management of prolactin-secreting pituitary adenomas: a Pituitary Society international consensus statement. Nat Rev Endocrinol 19(12):722–40 37670148 10.1038/s41574-023-00886-5

[9] Zamanipoor Najafabadi AH, Zandbergen IM, de Vries F, Broersen LHA, van den Akker-van Marle ME, Pereira AM et al (2020) Surgery as a viable alternative First-Line treatment for prolactinoma Patients. A systematic review and Meta-Analysis. J Clin Endocrinol Metab 105(3):e32–41 31665485 10.1210/clinem/dgz144 PMC7112976

[10] Bonneville JF, Bonneville F, Cattin F (2005) Magnetic resonance imaging of pituitary adenomas. Eur Radiol 15(3):543–548 15627195 10.1007/s00330-004-2531-x

[11] Jagannathan J, Sheehan JP, Jane JA Jr. (2007) Evaluation and management of Cushing syndrome in cases of negative sellar magnetic resonance imaging. Neurosurg Focus 23(3):E3 17961018 10.3171/foc.2007.23.3.4

[12] Tabarin A, Laurent F, Catargi B, Olivier-Puel F, Lescene R, Berge J et al (1998) Comparative evaluation of conventional and dynamic magnetic resonance imaging of the pituitary gland for the diagnosis of cushing’s disease. Clin Endocrinol (Oxf) 49(3):293–300 9861318 10.1046/j.1365-2265.1998.00541.x

[13] de Rotte AA, Groenewegen A, Rutgers DR, Witkamp T, Zelissen PM, Meijer FJ et al (2016) High resolution pituitary gland MRI at 7.0 tesla: a clinical evaluation in cushing’s disease. Eur Radiol 26(1):271–277 25991481 10.1007/s00330-015-3809-x PMC4666272

[14] Patel V, Liu CJ, Shiroishi MS, Hurth K, Carmichael JD, Zada G et al (2020) Ultra-high field magnetic resonance imaging for localization of corticotropin-secreting pituitary adenomas. Neuroradiology 62(8):1051–1054 32306052 10.1007/s00234-020-02431-x

[15] Durmuş ET, Atmaca A, Uzunkaya F, Çolak R, Durmuş B (2022) The lateralization accuracy of inferior petrosal sinus sampling in cushing’s disease: experiences of a tertiary center. Turk J Med Sci 52(5):1600–1608 36422478 10.55730/1300-0144.5500 PMC10395702

[16] Feng M, Liu Z, Liu X, Zhang X, Bao X, Yao Y et al (2018) Tumour lateralization in cushing’s disease by inferior petrosal sinus sampling with Desmopressin. Clin Endocrinol (Oxf) 88(2):251–257 29080355 10.1111/cen.13505

[17] Wind JJ, Lonser RR, Nieman LK, DeVroom HL, Chang R, Oldfield EH (2013) The lateralization accuracy of inferior petrosal sinus sampling in 501 patients with cushing’s disease. J Clin Endocrinol Metab 98(6):2285–2293 23553862 10.1210/jc.2012-3943 PMC3667263

[18] Kremer P, Forsting M, Ranaei G, Wüster C, Hamer J, Sartor K et al (2002) Magnetic resonance imaging after transsphenoidal surgery of clinically non-functional pituitary macroadenomas and its impact on detecting residual adenoma. Acta Neurochir (Wien) 144(5):433–43 12111499 10.1007/s007010200064

[19] Berkmann S, Roethlisberger M, Mueller B, Christ-Crain M, Mariani L, Nitzsche E et al (2021) Selective resection of cushing microadenoma guided by preoperative hybrid 18-fluoroethyl-L-tyrosine and 11-C-methionine PET/MRI. Pituitary 24(6):878–86 34155554 10.1007/s11102-021-01160-5

[20] Koulouri O, Steuwe A, Gillett D, Hoole AC, Powlson AS, Donnelly NA et al (2015) A role for 11C-methionine PET imaging in ACTH-dependent Cushing’s syndrome. Eur J Endocrinol 173(4):M107–20 26245763 10.1530/EJE-15-0616

[21] Haberbosch L, MacFarlane J, Koulouri O, Gillett D, Powlson AS, Oddy S et al (2024) Real-world experience with 11 C-methionine positron emission tomography in the management of acromegaly. Eur J Endocrinol 190(4):307–313 38482632 10.1093/ejendo/lvae028

[22] Bashari WA, van der Meulen M, MacFarlane J, Gillett D, Senanayake R, Serban L et al (2022) (11)C-methionine PET aids localization of microprolactinomas in patients with intolerance or resistance to dopamine agonist therapy. Pituitary 25(4):573–86 35608811 10.1007/s11102-022-01229-9 PMC9345820

[23] Bakker LEH, Verstegen MJT, Ghariq E, Verbist BM, Schutte PJ, Bashari WA et al (2022) Implementation of functional imaging using (11)C-methionine PET-CT co-registered with MRI for advanced surgical planning and decision making in prolactinoma surgery. Pituitary 25(4):587–601 35616762 10.1007/s11102-022-01230-2 PMC9345807

[24] Gillett D, Senanayake R, MacFarlane J, van der Meulen M, Koulouri O, Powlson AS et al (2022) Localization of TSH-secreting pituitary adenoma using 11C-methionine image subtraction. EJNMMI Res 12(1):26 35524902 10.1186/s13550-022-00899-7 PMC9079199

[25] Koulouri O, Kandasamy N, Hoole AC, Gillett D, Heard S, Powlson AS et al (2016) Successful treatment of residual pituitary adenoma in persistent acromegaly following localisation by 11C-methionine PET co-registered with MRI. Eur J Endocrinol 175(5):485–98 27562400 10.1530/EJE-16-0639

[26] Rodriguez-Barcelo S, Gutierrez-Cardo A, Dominguez-Paez M, Medina-Imbroda J, Romero-Moreno L, Arraez-Sanchez M (2014) Clinical usefulness of coregistered 11 C-methionine positron emission tomography/3-T magnetic resonance imaging at the follow-up of acromegaly. World Neurosurg 82(3–4):468–473 24239736 10.1016/j.wneu.2013.11.011

[27] Ishida A, Kaneko K, Minamimoto R, Hotta M, Inoshita N, Takano K et al (2023) Clinical decision-making based on 11 C-methionine PET in recurrent cushing’s disease with equivocal MRI findings. J Neurosurg 139(6):1671–1680 37410630 10.3171/2023.5.JNS23179

[28] Furnica RM, Devuyst F, Mathey C, Constantinescu SM, De Herdt C, Alexopoulou O et al (2025) Diagnostic value of 11 C-Methionine PET-CT imaging in persistent or recurrent Cushing’s disease after surgery. J Clin Endocrinol Metab 10.1210/clinem/dgaf047 39873396

[29] Tang BN, Levivier M, Heureux M, Wikler D, Massager N, Devriendt D et al (2006) 11 C-methionine PET for the diagnosis and management of recurrent pituitary adenomas. Eur J Nucl Med Mol Imaging 33(2):169–178 16228237 10.1007/s00259-005-1882-0

[30] Flaus A, Levigoureux E, Haesebaert J, Briet C, Castinetti F, Cristante J et al (2025) Prospective multicenter evaluation of [(11)C]Methionine PET/MRI sensitivity compared with MRI for localizing small pituitary neuroendocrine tumor or pituitary adenoma in Cushing disease. J Nucl Med. 10.2967/jnumed.124.269392 40841148 10.2967/jnumed.124.269392 PMC12487729

[31] Langen KJ, Hamacher K, Weckesser M, Floeth F, Stoffels G, Bauer D et al (2006) O-(2-[18F]fluoroethyl)-L-tyrosine: uptake mechanisms and clinical applications. Nucl Med Biol 33(3):287–94 16631076 10.1016/j.nucmedbio.2006.01.002

[32] Slagboom TNA, Stenvers DJ, van de Giessen E, Roosendaal SD, de Win MML, Bot JCJ et al (2023) Continuing challenges in the definitive diagnosis of Cushing’s disease: a structured review focusing on molecular imaging and a proposal for diagnostic work-up. J Clin Med. 10.3390/jcm12082919 37109254 10.3390/jcm12082919 PMC10144206

[33] Pruis IJ, Verburg FA, Balvers RK, Harteveld AA, Feelders RA, Vernooij MW et al (2024) [(18)F]FET PET/MRI: an accurate technique for detection of small functional pituitary tumors. J Nucl Med 65(5):688–92 38514085 10.2967/jnumed.123.266853

[34] Bakker LEH, Verstegen MJT, Manole DC, Lu H, Decramer TJM, Pelsma ICM et al (2024) (18)F-fluoro-ethyl-tyrosine PET co-registered with MRI in patients with persisting acromegaly. Clin Endocrinol (Oxf) 101(2):142–152 38818709 10.1111/cen.15079

[35] van Trigt VR, Bakker LEH, Lu H, Pelsma ICM, Verstegen MJT, van Furth WR et al (2024) Clinical use of [(18)F]fluoro-ethyl-L-tyrosine PET co-registered with MRI for localizing prolactinoma remnants. Pituitary 27(5):614–24 39042164 10.1007/s11102-024-01430-y PMC11513721

[36] Fleseriu M, Biller BMK, Freda PU, Gadelha MR, Giustina A, Katznelson L et al (2021) A pituitary society update to acromegaly management guidelines. Pituitary 24(1):1–13 33079318 10.1007/s11102-020-01091-7 PMC7864830

