# Supplementary material for: The clinical value of [18F]-fluoro-ethyl-L-tyrosine PET ([18F]FET-PET) correlated with MRI in patients with functioning pituitary adenomas: an observational cohort study
Source: Pituitary. 2026 Mar 7;29(2):46. doi: 10.1007/s11102-025-01634-w (PMC12967589; doi:10.1007/s11102-025-01634-w)
Supplement: Supplementary file 1 — (DOCX 72.4 KB) [file 11102_2025_1634_MOESM1_ESM.docx]

# Supplemental material

# The clinical value of [^18^F]-fluoro-ethyl-L-tyrosine PET ([^18^F]FET-PET) correlated with MRI in patients with functioning pituitary adenomas: an observational cohort study

L.S. van der Hoeven^1,2,3^, S.G. van Vugt^4^, T. Timmers^4^, E.A.M. Heshof^1^, M.S. Koopman^2,5^, E. Aronica^2,3,6^, J. Hoogmoed^2,3,7^, A.M. Pereira^1,2,3^, E. van de Giessen^2,4,8^, D.J. Stenvers^1,2,3^

^1^ Amsterdam UMC, University of Amsterdam, Department of Endocrinology and Metabolism, Amsterdam Gastroenterology Endocrinology and Metabolism (AGEM), Amsterdam, Netherlands;

^2^ Pituitary Center Amsterdam, Amsterdam, The Netherlands;

^3^ European Reference Network on Rare Endocrine Conditions (Endo-ERN), Amsterdam, the Netherlands

^4^ Department of Radiology & Nuclear Medicine, Amsterdam UMC, Vrije Universiteit, the Netherlands

^5^ Amsterdam UMC, University of Amsterdam, Department of Radiology and Nuclear Medicine, Amsterdam, the Netherlands;

^6^ Amsterdam UMC, University of Amsterdam, Department of (Neuro)Pathology, Amsterdam, The Netherlands;

^7^ Amsterdam UMC, University of Amsterdam, Department of Neurosurgery, Amsterdam, The Netherlands;

^8^ Amsterdam Neuroscience, Brain Imaging, Amsterdam, The Netherlands

**Corresponding author:** L.S. van der Hoeven, l.s.vanderhoeven@amsterdamumc.nl

**Supplemental Table 1. Definitions and cut-offs used for biochemical diagnosis, remission and recurrence of Cushing’s disease, acromegaly, prolactinoma and TSH-oma**

| Diagnosis | Criteria |
| --- | --- |
| Cushing’s disease | **Diagnosis/recurrence:** ACTH-dependent hypercortisolism with abnormal test results of at least 2 out of the 3 screening tests (overnight 1 mg DST (≥95 nmol/L), 24-hour UFC measurements (≥150 nmol/24 hours), and LNSC (≥4 nmol/L)). In case of suspected cyclic CD, the suspicion or diagnosis was based on expert opinion and, if needed, hair cortisol. A 7 mg DST (cortisol decrease ≥190 nmol/L or ≥50%) or IPSS, with CRH (dose: 100 µg) or desmopressin (dose: 10 µg) stimulation (depending on CRH availability), were both performed in 6 cases to confirm a pituitary origin of the hormonal hypersecretion. For IPSS, a central-to-peripheral ACTH ratio of >2.0 at baseline or >3.0 after stimulation with either CRH or desmopressin during IPSS was considered diagnostic for the presence of a corticotroph adenoma.  **Remission:** Immediate postoperative morning serum cortisol levels <137 mmol/L in combination with hydrocortisone dependency with or without hypercortisolism withdrawal symptoms, or the normalization of at least 2 out of 3 screening tests without the use of medication: 1 mg DST (<95 nmol/L), 24-hour UFC (< 150 nmol/24h), and LNSC (<4 nmol/L) with or without hypocortisolism. Medical-dependent biochemical remission was defined as normalization of 24-hour UFC (<150 nmol/24h) with the use of metyrapone. Hypocortisolism due to bilateral adrenalectomy was also considered biochemical remission. |
| Acromegaly | **Diagnosis/recurrence:** Abnormal OGTT (GH >0.4 mU/L), or, in case OGTT was not performed, IGF-1 level >2 SD in the presence of high clinical suspicion.  **Remission:** Postoperative normalization of the OGTT, or, in the absence of an OGTT, IGF-1 levels below 2.0 SD with or without medication |
| Prolactinoma | **Diagnosis/recurrence:** prolactin levels >1x ULN in the presence of symptoms of hyperprolactinemia.  **Remission:** PRL levels <1 ULN with or without medication |
| TSH-oma | **Diagnosis/recurrence:** Elevated fT4 with non-suppressed TSH levels.  **Remission:** fT4 levels within the normal range with or without medication**.** |

ACTH adrenocorticotropic hormone, CD Cushing’s disease, CRH Corticotropin-releasing hormone, DST dexamethasone suppression test, fT4 free thyroxine, GH growth hormone, IGF-1 insulin-like growth factor 1, IPSS inferior petrosal sinus sampling, LNSC late night salivary cortisol, OGTT oral glucose tolerance test, PRL serum prolactin, SD standard deviation, TSH thyroid-stimulating hormone, UFC urinary free cortisol, ULN upper limit of normal

**Supplemental Table 2. Data on disease stage and imaging results**

|  | **Sex** | **Age^a^** | **Disease stage** | **Pituitary surgery  before [^18^F]FET-PET** | **PA results of surgery  before [^18^F]FET-PET** | **Medication  discontinued <6 months prior to [^18^F]FET-PET** | **Medication used  during [^18^F]FET-PET** | **IPSS results** | **(Suspected) adenoma localization  based on MRI** | **Size of lesion  on MRI (mm)** | **Indication of [^18^F]FET-PET^b^** | **Localization based on [^18^F]FET-PET** | **Likelihood-scale of [^18^F]FET-PET^c^** | **Concordance between  [^18^F]FET-PET and MRI** |
| --- | --- | --- | --- | --- | --- | --- | --- | --- | --- | --- | --- | --- | --- | --- |
| **Cushing's disease** | | | | | | | | | | | | | | |
| Scan 1 | M | 57 | Diagnosis | 0 |  |  | Metyrapone 1000 mg/day |  | Left | <6 | 2 | Central | 3 | Discordant, MRI+/[^18^F]FET-PET+ different location |
| Scan 2 | F | 39 | Diagnosis | 0 |  |  |  | Pituitary origin - left | Right/central | <6 | 2 | Not visible | 1 | Discordant, MRI+/[^18^F]FET-PET- |
| Scan 3 | F | 58 | Diagnosis | 0 |  |  |  |  | Right | <6 | 2 | Right | 5 | Concordant positive |
| Scan 4 | M | 58 | Diagnosis | 0 |  |  |  |  | Right | <6 | 2 | Right | 5 | Concordant positive |
| Scan 5 | F | 45 | Diagnosis | 0 |  |  |  | Pituitary origin - left | Not visible |  | 1 | Left | 2 | Discordant,  MRI-/[^18^F]FET-PET+ |
| Scan 6 | F | 29 | Diagnosis | 0 |  |  | Metyrapone 500 mg/day | Pituitary origin – left^g^ | Right | <6 | 2 | Central | 3 | Discordant, MRI+/[^18^F]FET-PET+ different location |
| Scan 7 | F | 45 | Diagnosis | 0 |  |  |  | Pituitary origin^g^ | Not visible |  | 1 | Not visible | 1 | Concordant negative |
| Scan 8 | M | 42 | Diagnosis | 0 |  |  |  | Pituitary origin | Lesion 1: Right - caudal Lesion 2: Right - central | <6  <6 | 4 | Right central | 3 | Partly concordant |
| Scan 9 | F | 27 | Persistent disease | 2 | Corticotroph (CC) |  | Metyrapone 750 mg/day | Pituitary origin - right | Not visible |  | 1 | Right | 3 | Discordant,  MRI-/[^18^F]FET-PET+ |
| Scan 10^d^ | F | 45 | Persistent disease | 2 | Corticotroph (DG) |  |  |  | Not visible |  | 1 | Left | 3 | Discordant,  MRI-/[^18^F]FET-PET+ |
| Scan 11^e^ | F | 36 | Persistent disease | 3 | Corticotroph (DG) | Metyrapone 750 mg/day |  |  | Right/central | 6-9 | 3 | Right | 4 | Concordant positive |
| Scan 12 | F | 46 | Recurrence | 1 | NR |  |  |  | Left | <6 | 3 | Left | 5 | Concordant positive |
| Scan 13^d^ | F | 45 | Recurrence | 1 | Corticotroph (DG) |  |  |  | Not visible |  | 1 | Left | 3 | Discordant,  MRI-/[^18^F]FET-PET+ |
| Scan 14^e^ | F | 35 | Recurrence | 2 | Corticotroph (DG) |  |  |  | Central | 6-9 | 3 | Central | 4 | Concordant positive |
| Scan 15 | F | 25 | Recurrence | 1 | Corticotroph (CC) |  |  |  | Left | <6 | 3 | Left | 4 | Concordant positive |
| Scan 16 | F | 58 | Recurrence | 2 | Corticotroph |  |  |  | Left | <6 | 3 | Not visible | 1 | Discordant, MRI+/[^18^F]FET-PET- |
| Scan 17 | F | 33 | Recurrence | 2 | NR |  |  |  | Right | <6 | 3 | Not visible | 1 | Discordant, MRI+/[^18^F]FET-PET- |
| Scan 18 | F | 57 | Recurrence | 1 | Corticotroph (DG) |  |  |  | Right | ≥10 | 2 | Right | 5 | Concordant positive |
| Scan 19 | F | 55 | Other | 3 | NR |  | Hydrocortison 20 mg/day |  | Not visible |  | 6^±^ | Left | 4 | Discordant,  MRI-/[^18^F]FET-PET+ |
| **Acromegaly** | | | | | | | | | | | | | | |
| Scan 20 | F | 26 | Diagnosis | 0 |  |  |  |  | Central | <6 | 2 | Not visible | 1 | Discordant, MRI+/[^18^F]FET-PET- |
| Scan 21 | M | 53 | Diagnosis | 0 |  |  | Octreotide 20 mg/day |  | Not visible |  | 1 | Right | 2 | Discordant,  MRI-/[^18^F]FET-PET+ |
| Scan 22 | M | 20 | Persistent disease | 2 | Somatotroph (SG) |  | Lanreotide 120 mg/4 weeks |  | Lesion 1: Right Lesion 2: Left | ≥10 ≥10 | 4 | Right | 5 | Partly concordant |
| Scan 23^f^ | F | 35 | Persistent disease | 1 | Mammo-somatotroph | Lanreotide 160 mg/4 weeks |  |  | Not visible |  | 1 | Left | 5 | Discordant,  MRI-/[^18^F]FET-PET+ |
| Scan 24^f^ | F | 36 | Persistent disease | 2 | Mammo-somatotroph |  | Lanreotide 90 mg/4 weeks Pegvisomant 40 mg/week |  | Left | <6 | 3 | Left | 5 | Concordant positive |
| Scan 25 | M | 64 | Persistent disease | 1 | Gonadotroph |  |  |  | Left/central | ≥10 | 5, 6 | Left/central | 5 | Concordant positive |
| Scan 26 | F | 45 | Persistent disease | 2 | Somatotroph (DG) |  |  |  | Right/central | 6-9 | 2 | Right/central | 5 | Concordant positive |
| Scan 27 | M | 39 | Persistent disease | 3 + RTX | Somatotroph (SG) |  | Cabergoline 3 mg/week Lanreotide 40 mg/week Pegvisomant 30 mg/day |  | Lesion 1: Right sellar Lesion 2: Left posterior (tentorium) Lesion 3: Left (fissura orbitalis superior) | ≥10  6-9  ≥10 | 4 | Right | 5 | Partly concordant |
| Scan 28 | F | 61 | Recurrence | 1 | Somatotroph |  | Lanreotide 60 mg/week |  | Right | 6-9 | 3 | Right | 5 | Concordant positive |
| Scan 29 | F | 58 | Recurrence | 1 | Mammo-somatotroph |  |  |  | Right | 6-9 | 2 | Right | 5 | Concordant positive |
| Scan 30 | M | 29 | Recurrence | 2 | Somatotroph (DG) |  | Octreotide 10 mg/4 weeks |  | Central | ≥10 | 2 | Lesion 1: Central Lesion 2: Left | 5 | Partly concordant |
| Scan 31 | F | 60 | Recurrence | 1 | Somatotroph (SG) | Pegvisomant 60 mg/week |  |  | Right | <6 | 3 | Not visible | 1 | Discordant, MRI+/[^18^F]FET-PET- |
| Scan 32 | M | 38 | Recurrence | 1 | Somatotroph (DG) | Cabergoline 0.5 mg/week |  |  | Not visible |  | 1 | Left | 3 | Discordant,  MRI-/[^18^F]FET-PET+ |
| Scan 33 | M | 45 | Recurrence | 1 | Unknown | Lanreotide 120 mg/4 weeks |  |  | Left | 6-9 | 3 | Not visible | 1 | Discordant, MRI+/[^18^F]FET-PET- |
| **Prolactinoma** | | | | | | | | | | | | | | |
| Scan 34 | F | 22 | Diagnosis | 0 |  |  |  |  | Lesion 1: Right Lesion 2: Left | ≥10 6-9 | 4 | Right | 5 | Partly concordant |
| Scan 35 | M | 69 | Persistent disease | 1 | Lactotroph |  | Cabergoline 3.0 mg/week |  | Right | 6-9 | 3 | Right | 3 | Concordant positive |
| Scan 36 | F | 38 | Recurrence | 1 | Lactotroph |  | Cabergoline 1.5 mg/week |  | Left | 6-9 | 2 | Left | 5 | Concordant positive |
| **TSH-oma** | | | | | | | | | | | | | | |
| Scan 37 | M | 79 | Diagnosis | 0 |  |  | Thiamazole 10 mg/day |  | Right | 6-9 | 2 | Not visible | 1 | Discordant, MRI+/[^18^F]FET-PET- |

*CC* Crooks’ cell subtype, *DG* densely granulated, F female, [^18^F]*FET‐PET* [^18^F]fluoroethyl-L-tyrosine-PET, *IPSS* inferior petrosal sinus sampling, *M* male, *MRI* magnetic resonance imaging, *NR* no representative adenoma tissue, TSH*-oma* TSH-producing pituitary adenoma, *PA* histopathology, *RTX* radiotherapy, *SG* sparsely granulated

^a^ Age at the time of the [^18^F]FET‐PET

^b^ The indication of a [^18^F]FET‐PET was categorized as follows: 1) To identify and localize a lesion/remnant that has not been seen on MRI, 2) To confirm activity of a lesion/remnant, 3) To discriminate between an active remnant and postoperative changes, 4) To localize the most active lesion/remnant out of multiples lesions seen on MRI, 5) To localize the most active part of a remnant, and 6) Other

^c^ The assessments of the [^18^F]FET‐PETs were given a likelihood-scale: 1: no lesion visible, 2: likely no adenoma visible, 3: possibly adenoma visible, 4: likely adenoma visible, 5: certainly adenoma visible

^d^ Scan 11 and 14 are scans from the same patient

^e^ Scan 12 and 15 are scans from the same patient

^f^ Scan 23 and 24 are scans from the same patient

^g^ Performed after [^18^F]FET-PET

**Supplemental Table 3. Comparison of SUV values and Likelihood-scales between the PET/CT scanners**

|  | Ingenuity and Vereos (Philips)  n = 8 | Quadra (Siemens)  n = 20 | p-value |
| --- | --- | --- | --- |
| SUVmax of lesion | 2.6 [2.3-3.1] | 2.9 [2.6-3.2] | 0.309 |
| SUVpeak of lesion | 1.9 [1.5-2.2] | 1.6 [1.3-1.9] | 0.140 |
| SUVmax cavernous sinus | 2.6 ± 0.5 | 2.6 ± 0.4 | 0.671 |
| SUVpeak cavernous sinus | 1.9 ± 0.4 | 1.7 ± 0.3 | 0.427 |
| SUVmean temporal lobes | 1.0 ± 0.3 | 1.0 ± 0.2 | 0.936 |
| LBR^a^ | 2.8 [3.1-3.4] | 3.0 [2.5-3.2] | 0.722 |
| Likelihood-scale^b^ | 3.0 [1.0-5.0] | 4.0 [2.3-5.0] | 0.855 |

Data are presented as mean ± SD or median [1st-3rd IQR].

[^18^F]FET‐PET [^18^F]fluoroethyl-L-tyrosine-PET, LBR Lesion to background (temporal lobe) ratio, SUV Standardized Uptake Value

^a^ Lesion to background ratio (LBR) was calculated by dividing the SUVmax by the SUVmean of the left and right temporal lobe.

^b^ The assessments of the [^18^F]FET‐PETs were given a Likelihood-scale: 1: no lesion visible, 2: likely no adenoma visible, 3: possibly adenoma visible, 4: likely adenoma visible, 5: certainly adenoma visible

**Supplemental Table 4. Follow-up data**

|  | **Sex** | **Age^a^** | **Disease stage at time of  [^18^F]FET-PET** | **Follow-up duration (months)** | **Pituitary  surgery (n)** | **PA results first surgery  after [^18^F]FET-PET** | **Postoperative remission  after first surgery after [^18^F]FET-PET** | **PA results during further FU** | **Bilateral adrenalectomy /  development Nelson's syndrome** | **Medical therapy** | **Radio-therapy** | **Biochemical remission during further FU** | **Recurrence during FU** |
| --- | --- | --- | --- | --- | --- | --- | --- | --- | --- | --- | --- | --- | --- |
| **Cushing's disease** | | | | | | | | | | | | | |
| Scan 1 | M | 57 | Diagnosis |  | 1 | NR | Yes |  |  | 0 |  | Yes | No |
| Scan 2 | F | 39 | Diagnosis |  | 1 | NR | Yes |  |  | 0 |  | Yes | No |
| Scan 3 | F | 58 | Diagnosis |  | 1 | Corticotroph (DG) | Yes |  |  | 0 |  | Yes | No |
| Scan 4 | M | 58 | Diagnosis |  | 1 | NR | Yes |  |  | 0 |  | Yes | Yes |
| Scan 5 | F | 45 | Diagnosis |  | 2 | NR^f^ | No | NR | Yes / No | 1 |  | Yes^e^ | No |
| Scan 6 | F | 29 | Diagnosis |  | 1 | NR | Yes |  |  | 1 |  | Yes | No |
| Scan 7 | F | 45 | Diagnosis |  | 2 | NR | No | NR | Yes / No | 1 |  | Yes^e^ | No |
| Scan 8 | M | 42 | Diagnosis |  | 1 | Corticotroph | Yes |  |  | 0 |  | Yes | No |
| Scan 9 | F | 27 | Persistent disease |  | 1 | NR | No |  | Yes / Yes | 1 | Yes | Yes^e^ | No |
| Scan 10^b^ | F | 45 | Persistent disease |  | 0 |  |  |  |  | 1 |  | Yes^g^ | Yes |
| Scan 11^c^ | F | 36 | Persistent disease |  | 1 | NR | No |  |  | 0 |  | No | N/A |
| Scan 12 | F | 46 | Recurrence |  | 2 | Corticotroph (DG) | Yes | Corticotroph (DG) |  | 0 |  | Yes | Yes |
| Scan 13^b^ | F | 45 | Recurrence |  | 1 | Corticotroph (DG) | No |  |  | 1 |  | No | N/A |
| Scan 14^c^ | F | 35 | Recurrence |  | 2 | NR | No | NR |  | 1 |  | No | N/A |
| Scan 15 | F | 25 | Recurrence |  | 1 | NR | Yes |  |  | 0 |  | Yes | No |
| Scan 16 | F | 58 | Recurrence |  | 1 | NR | Yes |  |  | 1 |  | Yes | No |
| Scan 17 | F | 33 | Recurrence |  | 0 |  |  |  |  | 0 |  | Yes | No |
| Scan 18 | F | 57 | Recurrence |  | 1 | Corticotroph (DG) | Yes |  |  | 0 |  | Yes | No |
| Scan 19 | F | 55 | Other |  | 0 |  |  |  | Prior to [^18^F]FET-PET / Yes | 0 |  | N/A | N/A |
| **Acromegaly** | | | | | | | | | | | | | |
| Scan 20 | F | 26 | Diagnosis |  | 0^ⱡ^ |  |  |  |  | 2 |  | No | N/A |
| Scan 21 | M | 53 | Diagnosis |  | 0 |  |  |  |  | 1 |  | Yes^g^ | No |
| Scan 22 | M | 20 | Persistent disease |  | 1 | Somatotroph | Yes |  |  | 1 |  | Yes | No |
| Scan 23^d^ | F | 35 | Persistent disease |  | 1 | NR | No |  |  | 2 |  | Yes^g^ | No |
| Scan 24^d^ | F | 36 | Persistent disease |  | 0 |  |  |  |  | 2 |  | Yes^g^ | No |
| Scan 25 | M | 64 | Persistent disease |  | 0 |  |  |  |  | 1 |  | No | N/A |
| Scan 26 | F | 45 | Persistent disease |  | 0 |  |  |  |  | 0 |  | No | N/A |
| Scan 27 | M | 39 | Persistent disease |  | 0 |  |  |  |  | 3 |  | Yes^g^ | No |
| Scan 28 | F | 61 | Recurrence |  | 0 |  |  |  |  | 1 |  | Yes^g^ | No |
| Scan 29 | F | 58 | Recurrence |  | 1 | Mammo-somatotroph | Yes |  |  | 0 |  | Yes | No |
| Scan 30 | M | 29 | Recurrence |  | 0 |  |  |  |  | 1 |  | Yes^g^ | No |
| Scan 31 | F | 60 | Recurrence |  | 0 |  |  |  |  | 0 |  | No | N/A |
| Scan 32 | M | 38 | Recurrence |  | 0 |  |  |  |  | 1 |  | Yes^g^ | No |
| Scan 33 | M | 45 | Recurrence |  | 0 |  |  |  |  | 1 |  | Yes^g^ | Yes |
| **Prolactinoma** | | | | | | | | | | | | | |
| Scan 34 | F | 22 | Diagnosis |  | 1 | Lactotroph (DG) | Yes |  |  | 0 |  | Yes | No |
| Scan 35 | M | 69 | Persistent disease |  | 0 |  |  |  |  | 1 |  | No | N/A |
| Scan 36 | F | 38 | Recurrence |  | 0^h^ |  |  |  |  | 1 |  | No | N/A |
| **TSH-oma** | | | | | | | | | | | | | |
| Scan 37 | M | 79 | Diagnosis |  | 0 |  |  |  |  | 1 |  | Yes^g^ | Yes |

*DG* densely granulated, F female, [^18^F]*FET‐PET* [^18^F]fluoroethyl-L-tyrosine-PET, *M* male, *MRI* magnetic resonance imaging, *N/*A Not applicable, *NR* no representative adenoma tissue, *TSH-oma* TSH-producing pituitary adenoma, *PA* histopathology, *RTX* radiotherapy, *SG* sparsely granulated

^a^ Age at the time of the [^18^F]FET‐PET

^b^ Scan 11 and 14 are scans from the same patient

^c^ Scan 12 and 15 are scans from the same patient

^d^ Scan 23 and 24 are scans from the same patient

^e^ Remission after bilateral adrenalectomy

^f^ Histopathology results showed preexistent pituitary tissue with a possible focus of a corticotroph microadenoma, however, this was not sufficient for a confirmative histopathological result

^g^ Remission under medication (biochemical control)

^h^ Awaiting surgery

**Supplemental Table 5. Comparison of SUV values and Likelihood-scales between subgroups of positive [^18^F]FET-PETs**

|  | Surgical cohort – positive [^18^F]FET-PET | | | Overall cohort – positive [^18^F]FET-PET | | | | | | |
| --- | --- | --- | --- | --- | --- | --- | --- | --- | --- | --- |
|  | Confirmative histopathology and/or postop remission  (n = 12) | No confirmative histopathology and/or postop remission  (n = 5) | p-value | No treatment with pituitary-directed agents^a^  (n = 19) | Treatment with pituitary-directed agents^a^ during [^18^F]FET-PET  (n = 8) | Treatment with pituitary-directed agents^a^ discontinued within 6 months prior to [^18^F]FET-PET (n = 2) | p-value | Biochemical hypersecretion at latest assessment and positive [^18^F]FET-PET (n=25)^b^ | Biochemical control at latest assessment and positive [^18^F]FET-PET (n=3) | p-value |
| SUVmax of lesion | 2.9 [2.4-3.3] | 2.8 [2.5-3.1] | 0.712 | 2.8 [2.4-3.1] | 3.0 [2.1-3.6] | 2.7 (range 2.6-2.9) | 0.912 | 2.8 [2.5-3.1] | 3.3 (range 2.1-3.6) | 0.477 |
| SUVpeak of lesion | 1.5 [1.3-1.9] | 1.9 [1.5-2.3] | 0.292 | 1.6 [1.3-1.9] | 1.8 [1.4-1.9] | 1.9 (range 1.8-2.1) | 0.505 | 1.7 [1.4-1.9] | 1.3 (range 1.1-2.3) | 0.433 |
| LBR^c^ | 2.9 [2.4-3.4] | 2.2 [2.1-3.1] | 0.171 | 2.9 [2.2-3.2] | 3.0 [2.9-4.1] | 2.4 (range 2.1-2.7) | 0.098 | 2.9 [2.3-3.2] | 3.0 (range 3.0-4.1) | 0.248 |
| Likelihood-scale^d^ | 4.5 [3.3-5.0] | 3.0 [2.5-5.0] | 0.308 | 4.0 [3.0- 5.0] | 5.0 [4.3-5.0] | 4.0 (range 3.0-5.0) | 0.566 | 5.0 [3.0-5.0] | 4.0 (range 2.0-5.0) | 0.927 |

Data are presented as median [1st-3rd IQR] or median (range: minimum – maximum).

[^18^F]FET‐PET [^18^F]fluoroethyl-L-tyrosine-PET, LBR Lesion to background (temporal lobe) ratio, SUV Standardized Uptake Value

^a^ Pituitary-directed agents used in this cohort were somatostatin analogues (Octreotide and Lanreotide) and a dopamine receptor agonist (Cabergoline)

^b^ Scan 19 excluded in these calculations due to bilateral adrenalectomy before [^18^F]FET‐PET

^c^ Lesion to background ratio (LBR) was calculated by dividing the SUVmax by the SUVmean of the left and right temporal lobe.

^d^ The assessments of the [^18^F]FET‐PETs were given a Likelihood-scale: 1: no lesion visible, 2: likely no adenoma visible, 3: possibly adenoma visible, 4: likely adenoma visible, 5: certainly adenoma visible

**Supplemental Table 6. Pearson’s r and Spearman’s rho correlation coefficients between 24h UFC, IGF-1, and prolactin levels and SUV values and Likelihood-scale in positive [^18^F]FET-PETs**

|  | SUVmax of lesion | | SUVpeak of lesion | | LBR^a^ | | Likelihood-scale^a^ | |
| --- | --- | --- | --- | --- | --- | --- | --- | --- |
|  | rho | p-value | rho | p-value | rho | p-value | rho | p-value |
| *Cushing’s disease* | | | | | | | | |
| 24h UFC | -0.145 | 0.654 | 0.130 | 0.688 | 0.039 | 0.905 | -0.215 | -0.441 |
| *Acromegaly* | | | | | | | | |
| IGF-1 | -0.036 | 0.920 | 0.224 | 0.533 | -0.309 | 0.385 | 0.379 | 0.182 |
| *Prolactinoma* | | | | | | | | |
| PRL | 0.018 | 0.960 | 0.500 | 0.667 | -0.500 | 0.667 | -0.866 | 0.333 |

24 h UFC 24-hour urinary free cortisol, [^18^F]FET‐PET [^18^F]fluoroethyl-L-tyrosine-PET, IGF-1 insulin-like growth factor 1, LNSC late night salivary cortisol, LBR Lesion to background (temporal lobe) ratio, PRL serum prolactin, SUV Standardized Uptake Value

^a^ Lesion to background ratio (LBR) was calculated by dividing the SUVmax by the SUVmean of the left and right temporal lobe

^b^ The assessments of the [^18^F]FET‐PETs were given a Likelihood-scale: 1: no lesion visible, 2: likely no adenoma visible, 3: possibly adenoma visible, 4: likely adenoma visible, 5: certainly adenoma visible
